# Disparities by sex in care-seeking behaviors and treatment outcomes for pneumonia among children admitted to hospitals in Bangladesh

**DOI:** 10.1371/journal.pone.0213238

**Published:** 2019-03-07

**Authors:** Aliya Naheed, Robert F. Breiman, Md. Saimul Islam, Samir K. Saha, Ruchira Tabassum Naved

**Affiliations:** 1 Initiative for Non-Communicable Diseases, Health Systems and Population Studies Division, Dhaka, Bangladesh; 2 Health Systems and Infectious Diseases Division, Dhaka, Bangladesh; 3 Emory Global Health Institute, Emory University, Atlanta, Georgia, United States of America; 4 Child Health Research Foundation, Department of Microbiology, Dhaka Shishu Hospital, Dhaka, Bangladesh; 5 Universal Health Coverage, Health System and Population Studies Division, Dhaka, Bangladesh; University of Georgia, UNITED STATES

## Abstract

**Background:**

Incidence of community acquired pneumonia is high globally. In Bangladesh, more male children than female children are brought to hospitals for pneumonia. We examined if there was disparities in the severity of illness and outcome by sex among children who were admitted with pneumonia to hospitals in Bangladesh.

**Methods:**

Hospitalized children, aged 2 to 59 months, meeting a case definition of pneumonia were recruited in seven hospitals following parental consent. At baseline, study doctors obtained socio-demographic characteristics and care seeking behaviors for pneumonia, and then clinical data were collected throughout the hospital stay. Multivariate analysis was performed to determine if the sex of the child had a relationship with either illness severity on admission or outcome in the hospital.

**Results:**

Between May 2004 and December 2008, 6,856 children, including 35% females, were recruited. A total of 1,371 (19.9%) children had non-severe pneumonia, 4,118 (60.0%) had severe pneumonia, and 1,367 (19.9%) had very severe pneumonia. A higher proportion of hospitalized females had very severe pneumonia as compared to males (21.5% versus 19.1%; P = 0.01), but there was no difference by sex in the proportion of children with severe or non-severe pneumonia. There was no difference by sex observed in the clinical management provided in the hospital, but a greater proportion of females (4.7%) as compared to males (3.6%) died in hospitals (P = 0.04). In multivariate analyses, female sex was associated with very severe pneumonia on admission (OR: 1.26, 95% CI: 1.09–1.47) and fatal outcome in the hospitals (OR: 1.31, 95% CI: 1.01–1.71). Death in female children admitted with very severe pneumonia was 4 times higher than that reported in males (OR: 4.37, 95% CI: 3.24–5.89).

**Conclusion:**

Our data demonstrates a sex-based disparity in the severity of pneumonia and deaths among children admitted to hospitals in Bangladesh, despite no existing disparity by sex in hospital treatment. These findings call for further investigations to explore the determinants of health seeking behavior by parents with children with pneumonia in a community that favors males to females, and to understand the role of differences by sex in childhood pneumonia outcomes in Bangladesh.

## Introduction

Pneumonia is the leading cause of infant and childhood deaths worldwide [1]. In 2015, 5·9 million children under age five died globally; pneumonia attributed to 0·921 million (15.9%) of these deaths [2,3]. Multiple risk factors including bacterial etiologies, exposure to smoke from cooking, and severe malnutrition have been associated with deaths from community acquired pneumonia (CAP) among young children [4–7]. CAP in children has been substantially reduced in those countries that have achieved improvements in nutritional status and environmental conditions [8]. Introduction of Hib and pneumococcal vaccines have also attributed to substantial reduction of CAP in some countries [9–12]. However, many children in the poorer countries of Asia and Africa still do not receive these vaccines due to their prohibitively high cost, although the ecologic and other risk factors for pneumonia are still persisting in these countries [1,13]. In many settings, early detection and community based treatment of CAP with effective antibiotics, along with optimal clinical management of very severe pneumonia in hospitals, remain key tools to increase the chance of survival [14].

A recently published systematic review on hospital admissions for severe acute lower respiratory infections estimated that in 2010, 11.9 million (95% CI 10.3–13.9 million) episodes of severe acute lower respiratory infection and 3.0 million (2.1–4.2 million) episodes of very severe acute lower respiratory infections resulted in hospital admissions in children under five worldwide [15]. Pneumonia incidence is high among children in South Asia, and it is the leading cause of hospitalization among children under five years of age in Bangladesh [7,13,16]. In Bangladesh, the ratio of males to females among children under five years old is 1:1 [17]. Despite a higher incidence of hospital admission in male children than in female children, with the greatest disparity by sex being reported in Bangladesh and in other South Asian countries [18,19]. Until now there have been no published reports on sex-based disparities in the incidence of CAP in Bangladesh [20–24].

Parental preference for male children over female children when it comes to using health services, making choices on providers, decisions regarding hospitalization, and health expenditures have been consistently reported in studies from South Asia, including Bangladesh [25–35]. While data exists about the relationship between sex and pneumonia outcomes following hospitalization among adults in developed countries, [36,37] very little information is available regarding this relationship in children in developing countries. A previous report by our group suggests that male children are more frequently brought to tertiary care hospitals in Bangladesh for treatment [38]. However, that report was based on partial data obtained from a multi-year hospital surveillance and did not provide an insight into any disparities by sex for pneumonia admission. In this study we examined disparities in the severity of illness and in-hospital treatment outcomes by sex among children under five years in Bangladesh.

## Materials and methods

The study populations and methods of recruitment have been described in details previously [7,39]. Briefly, we conducted surveillance of suspected bacterial diseases among children under five years through a network of seven tertiary care teaching hospitals in Bangladesh. The hospitals were located in three different districts in Bangladesh: Dhaka, Chittagong, and Tangail district. Three were government hospitals, and three were private hospitals. Three private hospitals included the largest paediatric referral hospital in the country located in Dhaka city, one general hospital located in Chittagong city and one general hospital located in rural Tangail district 64.2 kilometer away from Dhaka [40]. A study physician was employed at each participating hospital six days a week (Saturday through Thursday) and reviewed admission records daily to identify children aged 2 to 59 months old. A child was considered eligible for recruitment, if met one of the case definitions for very severe pneumonia, severe pneumonia, or non-severe pneumonia using the modified case definitions of the World Health Organization (WHO)[41] as has been described below.

Very severe pneumonia: History of cough *or* difficulty breathing and at least one of the following danger signs: central cyanosis, inability to breastfeed or drink, vomiting everything, prostration/lethargy, severe respiratory distress/poor respiratory effort.
Severe pneumonia: History of cough *or* difficulty breathing with chest in drawing, but no danger signs.
Non-severe pneumonia: History of cough *or* difficulty breathing with fast breathing (>60 breaths/min if aged <2 months; >50 breaths/min if aged 2–11 months; >40 breaths/min if aged 12 months to <60 months) but no chest in drawing or danger signs.

Children who met one of the eligibility criteria and had a blood specimen obtained for culture were recruited following obtaining a written voluntary consent of their parents/caretaker who were present with the child in the hospital [7]. The study physicians used a pre-tested, standardized case report form to collect data from the parents, including age and sex of the child, the parents’ education level, and if the child was brought to the hospital prior to consulting with a doctor or not. If a child was treated with an antibiotic prior to hospitalization, the study physicians inquired about the specific brand name or generic name of the antibiotic and the specific dosage administered to the child at home, then verified the information by either cross checking with a prescription or left over medicine, or with any supporting documents brought by the parents.

The study physicians conducted a physical examination for each child and collected data including the time of illness onset, signs and symptoms, duration of treatment before hospitalization if any, and treatment received in the hospital throughout the hospitalization period. The study physicians assessed the weight of the child and assessed the severity of pneumonia following a [41]protocol-specific algorithm. They also collected clinical data systematically from the hospital records, including the programmatic clinical management received during hospitalization and antibiotics prescribed by the hospital doctors to children at doses adjusted for age and weight as per the standard antibiotic treatment guideline until one of the following outcomes were met: discharge, referral, left against a medical advice, or death.

### Data analysis

The main outcome of interest was severity of illness defined as ‘very severe pneumonia’, ‘severe pneumonia’ and ‘non-severe pneumonia’. Child’s age was collected in a single year between 2 months and 59 months, and categorized into ‘infant’ (2–11 months of age) or ‘older’ (12–59 months of age). Parental education was defined as the highest level of academia completed and then categorized into ‘no formal schooling’ (never went to school), ‘less than primary’ (1–4 years of schooling), ‘completed primary’ (5–10 years of schooling), and ‘completed >10 years of schooling’ (11 or more years of schooling). Nutritional status was measured using the anthropometric indicator of weight-for-age in the form of Z-score (WAZ), using the World Health Organization (WHO) Anthro 2006 software [42]. The Z-score depicted the deviation from the median weight of the child according to the WHO reference of the median of the standard growth curve. Underweight was defined as a Z-score less than -2 standard deviation (SD), severely underweight was defined as a Z-score less than -3 SD, and other children who did not fall under those categories were defined as normal weight [43]. Length of stay (LOS) was defined as the number of days admitted in the hospital. Outcomes in the hospital were defined as ‘improved’ (if a child was cured at the time of discharge), ‘referred’ (if a child was transferred to another facility for better treatment), ‘left against medical advice’ (if a child was discharged despite warning of risk from medical personnel or absconded from the hospital without the knowledge of the hospital staff), or ‘fatal outcome’ (if a child died in hospital after enrollment in the study). Blood culture status of a child was a ‘positive blood culture’ if a bacterium was isolated in blood culture.

Data were entered using Fox Pro (version 2.6), and analyzed using SPSS version 20 (SPSS Inc. Released 2009. PASW Statistics for Windows, Version 20.0. Chicago: SPSS Inc). Frequency distribution was used to summarize the categorical variables, mean and standard deviation (SD) were used to report the continuous variables of normally distributed data, and median and interquartile range were reported for the non-normal data. Bivariate analyses were performed to detect differences by sex at the time of hospitalization, treatment provided in the hospital and the outcomes in the hospital. Finally, multivariate analyses were conducted to identify if sex had contributed to very severe pneumonia or severe pneumonia on admission compared to non-severe pneumonia (logistic regression), and fatal outcome in hospital compared to other than a ‘fatal outcome’ (logistic regression) after adjusting for those variables that were found to be statistically significantly different (as determined by a p-value of 0.05) in bivariate analyses. Variables adjusted for multivariate analysis included age and sex of the child, father’s education, history of antibiotic use prior to hospitalization, parents’ consultation with a doctor prior to hospitalization of the child, nutritional status, admission to a government or private hospital, duration between illness onset and hospital admission, and positive blood culture. T-tests or Mood’s Median test were applied to compare means or medians, respectively, across two groups. Pearson’s chi-square test was applied to compare the frequency of an event occurrence across two groups. A few variables had a missing value for a few case records ranging between 0.1% and 2% cases, and those case records were excluded at the time of analyses of that variable. Bivariate and multivariable analyses were applied for assessing measures of association, and were reported as odds ratios (OR) with a 95% confidence interval (CI). Statistical significance was set at p<0.05.

### Ethics

The study was approved by the Ethical Review Committee (ERC) of the International Centre for Diarrhoeal Disease Research, Bangladesh (icddr,b). Written informed consent was obtained from the parents or caretakers of the children prior to their recruitment into the study.

## Results

Between May 2004 and December 2008, 80,846 children were admitted to seven hospitals; 41,414 children (51.2%) had a blood culture done, and parents/caregivers of 38,805 (93.7%) children were approached for a written voluntary consent. Refusal to participate was low at 1.4%, leaving 38,259 children to be enrolled in the study. Among those enrolled, 6,856 (17.9%) children met the case definition of pneumonia according to the modified case definitions of the WHO and were included in the analyses.

The mean (±SD) age of the children with pneumonia was 10.1 (±10.0) months and 35.0% were females (Table 1). Males were significantly younger than females (9.6 (±9.6) versus 11.1 (±10.5), respectively; P<0.001). No education or less than primary education was reported in mothers of 1,912 (27.9%) children and in fathers of 1,863 (27.2%) children, while 286 (4.2%) mothers and 712 (10.4%) fathers reported to have completed more than 10 years of schooling (Table 1). Parental education was similar across all children, except that the proportion of fathers who had completed more than 10 years of schooling was higher among the female children than the male children (279[11.9%] vs. 433[9.9%], respectively; P = 0.03).

**Table 1:** Characteristics of children with pneumonia at the time of hospital admission and difference by sex.

Characteristics	Total (N = 6856)	Female (N = 2401)	Male (N = 4455)	P value^$^
Child age (Mean ± SD)	10.11±9.95	11.06±10.52	9.59±9.59	p<0.001^#^*
Child age category				
^a^Infant (2–11 month)	4871 (71.1)	1598 (66.6)	3273 (73.5)	0.001*
Older (12–59 month)	1985 (28.1)	803 (33.4)	1182 (26.5)	
Mother’s education, *n (%)*^b^				
No formal schooling	1912 (27.9)	654(27.2)	1258(28.2)	Ref.
Less than primary	936 (13.7)	324(13.5)	612(13.7)	0.83
Completed primary	3715 (54.2)	1308(54.5)	2407(54.0)	0.46
Completed > 10 years of schooling	286 (4.2)	113(4.7)	173(3.9)	0.08
Father’s education, *n(%)*^c^				
No formal schooling	1863 (27.2)	614(25.6)	1249(28.0)	Ref
Less than primary	675 (9.9)	243(10.1)	432(9.7)	0.15
Completed primary	3598 (52.5)	1262(52.6)	2336(52.4)	0.12
Completed > 10 years of schooling	712 (10.4)	279(11.6)	433(9.7)	0.03*
Overall severity of illness on admission, *n (%)*				
Non-severe pneumonia	1371(20.0)	453 (18.9)	918 (20.6)	Ref
Severe pneumonia	4118 (60.1)	1432 (59.6)	2686 (60.3)	0.25
Very severe pneumonia	1367 (19.9)	516 (21.5)	851 (19.1)	0.01*
Father completed > 10 years of schooling, *n (%)*				
Non-severe pneumonia	173(24.3)	62(22.2)	111(25.6)	Ref.
Severe pneumonia	390(54.8)	161(57.7)	229(52.9)	0.22
Very severe pneumonia	149(20.9)	56(20.1)	93(21.5)	0.74
History of antibiotic within 5 days before admission, (*n = 5458*,%)	3077 (56.4)	2022(57.3)	1055(54.7)	0.06
Non-severe pneumonia	583 (18.95)	193(18.3)	390(19.3)	Ref.
Severe pneumonia	1929 (62.69)	664(62.9)	1265(62.6)	0.55
Very severe pneumonia	565 (18.36)	198(18.8)	367(18.2)	0.48
Duration between illness onset and admission, median (interquartile range) days	4 (3,6)	4(3,6)	4(3,6)	0.33**
Parents brought children without consulting with a doctor, *n(%)*	3217 (46.9)	1163(48.4)	2054(46.1)	0.06
Non-severe pneumonia	882 (27.42)	286(24.6)	596(29.0)	Ref.
Severe pneumonia	1766 (54.9)	635(54.6)	1131(55.1)	0.07
Very severe pneumonia	569 (17.69)	242(20.8)	327(15.9)	0.01*
Parents brought children after consulting with a doctor^d^, *n(%)*	3639 (53.1)	1238(51.6)	2401(53.9)	0.06
Non-severe pneumonia	489 (13.44)	167(13.5)	322(13.4)	Ref.
Severe pneumonia	2352 (64.63)	797(64.4)	1555(64.8)	0.91
Very severe pneumonia	798 (21.93)	274(22.1)	524(21.8)	0.97
Nutritional status ^e^, *n (%)*				
Normal (waz ≥ -1 SD)	1600 (23.6)	590(24.9)	1010(22.9)	Ref
Mildly underweight (<-1 SD waz ≥ -2 SD)	1721(25.4)	616(26.0)	1105(25.1)	0.51
Moderately underweight (<-2 SD waz ≥-3 SD)	1645 (24.3)	557(23.5)	1088(24.7)	0.07
Severe underweight (waz <-3 SD)	1810 (26.7)	604(25.5)	1206(27.4)	0.03*
Children admitted in a government hospital, *n(%)*	4031(58.8)	1419(59.1)	2612(58.6)	0.70
Non-severe pneumonia	549 (13.6)	196(13.8)	353(13.5)	Ref.
Severe pneumonia	2712 (67.3)	940(66.2)	1772(67.8)	0.64
Very severe pneumonia	770 (19.1)	283(19.9)	487(18.6)	0.69
Children admitted in a private hospital, *n(%)*	2825 (41.2)	982(40.9)	1843(41.4)	0.70
Non-severe pneumonia	822 (29.1)	257(26.2)	565(30.7)	Ref.
Severe pneumonia	1406 (49.8)	492(50.1)	914(49.6)	0.07
Very severe pneumonia	597 (21.1)	233(23.7)	364(19.7)	0.02*

^a^Infant: Child less than 12 months of age.

^b^ Data missing for 7 children.

^c^ Data missing for 9 children.

^d^ Three female and one male were referred by the health workers in the community. The remaining 1235 female and 2400 male were brought to hospital by parents after consulting with a doctor.

^$^ All comparisons are made using chi-squared tests unless otherwise noted

** Mood’s Median Test

^#^ Independent sample t-test

^e^ 80 sample were missing and the denominator was 6776.

* Statistical significance at p<0.05

The median duration between illness onset and admission was 4 days (IQR:3–6) and 3,077 (56%) children received an antibiotic before hospitalization. A pathogenic bacterium was isolated from blood culture in 314 (4.6%) children suggesting bacterial infection. About 26.7% of all children were severely underweight (weight for age Z-score <-3 SD). More females than males were severely underweight (1206[27.4%] vs. 604[25.5%], respectively; p = 0.03).

According to the case definitions of pneumonia, 1,371 (20.0%) children had non-severe pneumonia, 4,118 (60.1%) had severe pneumonia, and 1,367 (19.9%) had very severe pneumonia at admission. A higher proportion of the females than the males had a very severe pneumonia (516[21.4%] vs. 851[19.1%], respectively; P = 0.01). After consulting with a medical doctor, 3,639 (53.0%) children were brought to the hospitals (Table 1). There was an observed difference across the sex of the child in the number of children admitted with very severe pneumonia when parents brought their child to the hospital without first consulting a medical doctor (242[20.8%] vs. 327[15.9%], respectively; P = 0.01). Of the 2,825 (41%) children who were admitted to private hospitals, more females than males had very severe pneumonia at admission (233[23.7%] vs. 364[19.7%], respectively; P = 0.02). (Table 1)

In the bivariate analyses, several factors were found to have contributed to very severe pneumonia in children on admission compared with severe or non-severe pneumonia. The odds of hospital admissions with very severe pneumonia was four times higher among children who were severely underweight than children with normal weight or who were moderately underweight (OR: 4.25; 95% CI: 3.74–4.82) (Table 2). Hospital admissions due to very severe pneumonia were 76% higher among infants than in older children (OR: 1.76; 95% CI: 1.52–2.03); 60% higher among those who had a positive blood culture (OR:1.60; 95% CI: 1.24–2.06) than who did not have a positive blood culture; 31% higher in children who were brought to the hospital after consulting with a doctor (OR: 1.31; 95% CI: 1.16–1.47); 16% higher among females than males (OR: 1.16; 95% CI: 1.03–1.31); 19% higher for children who used antibiotics prior to hospitalization (OR: 1.19; 95% CI: 1.04–1.36); and 14% higher among children who were admitted to a private hospital compared to those admitted to a government hospital (OR: 1.14; 95% CI: 1.01–1.28). Females were found to have higher odds of being admitted with very severe pneumonia than males (OR: 1.26, 95% CI: 1.09–1.47) after adjusting for age group, antibiotics prior to hospitalization, positive blood cultures, being severely underweight, being admitted to a private hospital, and whose parents brought their children after consulting with a doctor prior to hospitalization.

**Table 2:** Risk factors for very severe pneumonia in children at time of hospital admission.

Variables	Very severe pneumonia n = 1367(%)	Severe or non-sever pneumonia N = 5489 (%)	Unadjusted OR ^a^ (95% CI)	Adjusted OR ^a^ (95% CI)
Sex				
Female	516 (21.5)	1885 (34.3)	1.16 (1.03–1.31)*	1.26 (1.09–1.47)*
Male	851 (19.1)	3604 (65.7)	Ref.	Ref.
Child age				
Infant (2–11 month)	1088 (22.3)	3783 (68.9)	1.76(1.52–2.03)*	1.72(1.44–2.04)*
Older (12–59 month)	279 (14.1)	1706 (31.1)	Ref.	Ref.
Father education, n(%)^c^				
Complete >10 yrs of schooling	149 (20.9)	563 (10.3)	1.07(0.88–1.30)	
Did not complete >10 yrs of schooling	1217 (19.8)	4919 (89.7)	Ref.	Not included
Yes	565 (18.4)	2512 (57.2)	Ref.	Ref.
No	502 (21.1)	1879 (42.8)	1.19(1.04–1.36)*	1.20(1.04–1.40)*
Parents brought children to hospital				
Without consulting with a doctor	569 (17.7)	2648 (48.2)	Ref.	Ref.
After consulting with a doctor	798 (21.9)	2841 (51.8)	1.31(1.16–1.47)*	1.26(1.08–1.46)*
Nutritional status				
Severe underweight (waz < -3SD)	677 (37.4)	1133 (20.6)	4.25(3.74–4.82)*	4.15(3.58–4.81)*
Others group	612 (12.3)	4354 (79.4)	Ref.	Ref.
Children admitted in-				
Government	770 (19.1)	3261 (59.4)	Ref.	Ref.
Private	597 (21.1)	2228 (40.6)	1.14(1.01–1.28)*	1.71(1.47–1.99)*
Blood culture report				
Positive	88 (28.0)	226 (4.1)	1.60(1.24–2.06)*	1.36(0.99–1.88)
Negative	1279 (19.6)	5263 (95.9)	Ref.	Ref.

^a^ Odds Ratio.

^c^ Data missing for 9 children

*Statistical significance at p<0.05.

At discharge 3,890 children were diagnosed as having pneumonia (56.7%) by the hospital doctors. Other ailments that children were also diagnosed with by the hospital doctors at discharge included other respiratory diseases (2,172 [31.6%]), sepsis or meningitis (395 [6%]) had, seizures or convulsion disorders (96 [1.4%]), and other various diseases (292 [4.2%]). The majority of the children were treated with an antibiotic 6,818 (98%) after being admitted in hospitals, and females were more frequently treated with an injectable antibiotic over an oral antibiotic as compared to males (2216[92%] vs. 4045[91%], respectively, P = 0.03); although the difference was not significant after being adjusted for the severity of illness (P = 0.12) (S1 Table). Sixty nine percent children were prescribed two or more antibiotics. On average, children received an antibiotic for 4.8(±2.5) days in the hospitals and the duration of antibiotic treatment did not differ by sex (S1 Table).

Almost all children 6,717 (97.9%) were provided with a supportive measure while being treated with an antibiotic in the hospitals, including 5,118 (75.7%) with nebulization, 4,480 (67.1%) with antipyretic medicine, 4,405 (65.6%) with oxygen inhalation, 3,298 (49.1%) with a bronchodilator, and 305 (4.7%) received special care, such as that at an intensive care unit. The duration of the hospital stay was 5±3.5 days. There was no difference by sex in the treatment provided in the hospitals (S1 Table). Illness resolved prior to hospital discharge in 5,560 (81.1%) of children, whereas 40 (0.6%) were referred to another hospital for better management and 986 (14.1%) left the hospital against the medical advice of a clinician. No sex disparity was observed in outcome among those children who were alive till the end of their hospital stay (S1 Table).

In total, 276 of children died in the hospital, resulting in an overall case fatality rate (CFR) of 3.6% (S1 Table). The CFR was higher among females (4.8%) than males (3.6%); P = 0.04, and CFR in females among infants was higher than males (5.8% in females vs. 4.1% in males, p = 0.01). CFR was also higher in those children who were infant, whose father did not complete >10 years of schooling, who were brought to the hospital after consulting with a doctor, who suffered from severe malnutrition, who had a positive blood culture, who had received injectable antibiotics, and who had a very severe pneumonia at the time of admission (S2 Table). Half of the children who died in the hospital did so within 5 (IQR: 3–10) days of admission and death in the hospital was positively correlated with a delay from illness onset to hospital admission (OR: 1.14, 95% CI: 1.04–1.12), although no difference by sex was observed (P = 0.26).

After adjusting for all other variables found to have an association with death in the hospital in multivariate analyses we found that females had higher odds of death in the hospital compared to males (OR: 1.31, 95% CI: 1.01–1.71) (Table 3, Model 1). Overall, children who were admitted to the hospital with very severe pneumonia had 6 times higher odds of death (OR: 6.52, 95% CI: 4.93–8.61) than those who had a severe pneumonia or non-severe pneumonia at the time of admission (Table 3, Model 1), and sex of the child was borderline significant (P = 0.05). However, the females admitted with very severe pneumonia had 4 times higher odds of death in the hospital (OR: 4.37, 95% CI: 3.23–5.90) than other females after adjusting for all other variables associated with death in the hospital in bivariate analyses. (Table 3, Model 2).

**Table 3:** Risk factors for a fatal outcome for children with pneumonia in the hospital.

Factors	Model-1: Without interaction term		Model-2: With interaction term	
	Beta(SE)	OR(95% CI)	Beta(SE)	OR(95% CI)
Female child(Ref: male)	0.27(0.14)	1.31(1.01–1.71)*	-	-
Infant (Ref: older child; 12–59 month)	0.40(0.17)	1.50 (1.06–2.09)*	0.55(0.17)	1.73 (1.25–2.41)*
Father did not complete >10 years of schooling(Ref: >10 yrs of schooling)	0.57(0.27)	1.77 (1.04–2.99)*	0.42(0.27)	1.53 (0.91–2.58)
Parent brought children consulting with a doctor (Ref: Without consulting with a doctor)	0.09(0.13)	1.10 (0.84–1.42)	0.14(0.13)	1.14 (0.88–1.48)
Severe underweight(waz<-3) (Ref: Others group)	0.57(0.14)	1.79 (1.36–2.35)^#^	0.94(0.13)	2.57 (1.98–3.34)*
Blood culture positive (Ref: blood culture negative)	0.67(0.24)	1.94 (1.22–3.08)*	0.67(0.23)	1.96 (1.24–3.09)*
Taken injectable drug(Ref: had not taken)	1.09(0.46)	2.94 (1.19–7.27)*	1.13(0.46)	3.09 (1.26–7.61)*
Very severe pneumonia(Ref: severe pneumonia or non- severe)	1.88(0.14)	6.52 (4.93–8.61)^#^	-	-
Length of hospitalization (LOS)	0.002 (0.001)	1.30 (1.08–1.56)*	0.003 (0.001)	1.30 (1.09–1.55)*
Female children with very severe pneumonia ^a^	-	-	1.47(0.15)	4.37 (3.23–5.90)

^a^ Interaction term;

Statistical significance at p-value <0.05* and P<0.00^#^

OR = Odds Ratio

## Discussion

Our study suggests that parents are more likely to bring their male children to the hospital for pneumonia than their female children and that female children suffer from more severe pneumonia than male children when they are admitted to the hospital. Our study has also documented that the risk of death from pneumonia is higher among females than males, and that female children with very severe pneumonia are four times more likely to die than males and other females despite no sex disparity in treatment in the hospital. This suggests a sex disparity in parent’s hospital seeking behaviors for their female children with pneumonia and a sex disparity in pneumonia outcomes in hospitals in Bangladesh.

We observed that at a younger age, more males than females were brought to the hospitals by their parents or caregiver, and that among older children, more females than males had very severe pneumonia on admission. Since our observations with the children were limited to the period of hospitalization, it was not possible to investigate what community-level factors were associated with parents bringing their children to hospital or why we observed a disparity in the severity of illness on admission by sex. There may be a biological plausibility that very severe pneumonia is more common among older female children in the community, leading to an increased hospitalization of females with very severe pneumonia. However, evidence from Bangladesh does not support that there has been a sex disparity in the severity of pneumonia for children beyond infancy,[44] so we can exclude this possibility. But in this study we observed that more female than male children had very severe pneumonia at hospital admission when they were brought by their parents without consulting with a doctor first. This sex disparity was not observed for those children who were brought to the hospital after consulting with a doctor. If the latter observation is representative of the larger population, it is possible that more young females with a very severe illness might have already died at home without their parents having had sought care from a medical doctor. Or, perhaps the parents chose not to bring their female children with pneumonia to the hospital and they were treated elsewhere, presumably by an untrained provider. Or, females may only be brought to hospital when they have a more severe form of disease.

We observed that a higher proportion of fathers of female children admitted to the hospital for pneumonia had completed >10 years of schooling as compared to fathers of male children, and that the mothers’ education did not vary across males and females who were admitted. It is possible that fathers with higher education bring their ill daughters to the hospitals more frequently than fathers who are less educated. Further, females seem to need to have a more severe level of disease before an educated father brings the child to the hospital. This differs from what is observed with males, where males are brought to the hospital irrespective of disease severity or age. This indicates that parents may show preference towards their male children when making the decision for hospitalization for pneumonia. Evidence in Bangladesh suggests that only 37% of children with pneumonia are brought to a trained provider for treatment. Thus, our observation of more severe pneumonia in older females in the hospitals can be explained by it being a low priority in the community to bring females to the hospital when they have pneumonia [45]. This overall phenomenon indicates a need to improve health literacy in the community to raise awareness about the severity of pneumonia and the importance of bringing all children to the hospital for care.

Deaths due to pneumonia in children under five years of age can be reduced by 70% if treated with effective antibiotics [14]. This study shows that about half of children received an antibiotic within five days of hospital admission. Although this study does not demonstrate a disparity by sex in the treatment of children with pneumonia in hospitals, it does show that female children are prescribed more injectable antibiotics than the males, which is likely due to the severity of illness in females at the time of admission. Although a large majority of the children required oxygen inhalation and other supportive care in addition to antibiotics, most of the children improved following their treatment irrespective of the sex of the child. We also did not observe a difference in the occurrence of bacterial infection in children with pneumonia by sex, but did observe that children who were not treated with antibiotics prior to hospitalization had more severe pneumonia than other children. This study demonstrates that despite any observed disparity by sex in treatment at the hospital, the risk of death in the hospital was high for females when admitted with very severe pneumonia. A systematic review of 37 hospital based studies conducted in high-income countries reported that female children who were admitted to the hospital with severe acute lower respiratory tract infections had a higher case fatality than males in infancy [15]. Our analysis does not support the notion that there are within hospital differences in care by sex, but instead suggest that this disparity may be due to care-seeking behaviors of the parents of children with pneumonia prior to hospitalization. The overall study findings indicate that early referral to hospitals for females with very severe pneumonia may optimize their clinical management and reduce observed disparities in fatal outcomes for pneumonia by sex in hospitals in Bangladesh.

Our study also identified having very severe pneumonia as a risk factor for high risk of death in children, while infancy, severe malnutrition, and bacterial infection were identified as predictors for having very severe pneumonia at the time of hospital admission. Evidence of South Asian countries reported that the children who were hospitalized with severe or very severe pneumonia had higher risk of fatal outcomes compared with the non-severe children [46,47]. Further, low literacy of fathers proved to be a significant contributor to overall death in children, supporting a previously reported inverse relationship between the number of years of a fathers’ education and childhood death [48]. Although this study suggests a small difference in childhood death by sex, it is significant and raises broader concerns regarding multiple risk factors associated with death from community acquired pneumonia in children in Bangladesh.

The recent WHO guidelines recommend community-based case management for pneumonia when danger signs such as central cyanosis, inability to breastfeed or drink, vomiting everything are absent [49]. Our findings of more severe pneumonia in females at the time of hospital admission indirectly suggest a lack of community-based case management for pneumonia. This supports efforts to strengthen community-based services for childhood illness, such as community based IMCI (Integrated Management of Childhood Illnesses) and integrated community case management (ICCM) for pneumonia [50,51], to improve access to pneumonia treatment, particularly for younger females who are less likely to be brought to facilities unless the illness is more severe. A recently published systematic review confirms that severe malnutrition increases the risk of death from pneumonia among children younger than 5 years old, and frequency and spectrum of bacterial infection may be different from those who do not have severe malnutrition [4]. We observed that more than a quarter of the children with pneumonia had severe malnutrition, with the males being inflicted with more severe malnutrition than females at the time of admission. However, it is concerning that female children hospitalized for pneumonia are at higher risk of having more severe pneumonia, despite their better nutritional status. This may be due to a lack of immunization, which has been identified as a risk factor for pneumonia in young children [52], and sex discrimination in immunization coverage has previously been reported in Bangladesh [53]. Malnutrition is substantial in children under the age of five in Bangladesh, indicating that improvement in child nutrition in the community, along with comprehensive immunization coverage, can be essential to increase the chance of survival for children with pneumonia in hospitals [54]. The overall situation demands a compelling need for early detection of CAP in the community and strengthening primary care for pneumonia, including prevention.

While there is limited evidence to support parental preference for male children when deciding to seek care for pneumonia in Bangladesh, our findings are supported by studies in other countries that had similar findings. One study, conducted in rural India among mothers with children under five years old, reported that females with diarrhoea are less frequently treated by the qualified doctors as compared to males. The same study documented a significant delay in hospitalization and a smaller healthcare expenditure for females than males [24]. A similar situation has also been reported in Egyptian children for treating diarrhea in rural settings; fathers played an important role in obtaining services from a qualified health professional [55]. A study conducted in the Rural Uttar Pradesh, India also reported that care-seeking for female children is neglected compared to male children [56]. However, in Kerala, India, where utilization rate of receiving medical service is high in the middle-income group, disparities by sex were not observed in care-seeking behaviors for acute respiratory tract infections or diarrhea. However, there was a notable disparity in utilizing modern medical care as opposed to an alternative health care across urban and rural areas and high and low social classes [57]. One study in Pakistan reported more deaths in females than male infants who were admitted to the hospital, however, there was no difference across sex in careseeking patterns of parents or in healthcare expenditures [58]. On the contrary, while there is limited evidence in Vietnam to support any disparities in health-seeking behaviors by sex, the duration of hospitalization for both diarrhea and pneumonia is longer for males than females [59]. Despite limited evidence, there is a trend that indicates parental preference for male children when seeking care for pneumonia in Asia. We tend to believe that the findings from our study possibly corroborate a common phenomenon in other countries in South Asia.

While this study identifies a gap in parents seeking hospitalized care for their female children with very severe pneumonia, there are some limitations that may influence our findings. First, this study was conducted in tertiary-care hospitals, so we do not know whether sex-based differences in hospital attendance reflect actual sex-based differences in the incidence of pneumonia or the severity of pneumonia in the community. Without an evidential proof it is difficult to infer that observing a higher proportion of males being brought to the hospital with pneumonia is due to parents showing preferential treatment of their male children and seeking care more often for them.

Second, in this study we observed a disparity in the severity of illness at the time of hospital admission by sex among children whose parents did not consult a doctor prior to bringing them to the hospital. This suggests a lack of access to qualified providers in the community who may have contributed to the observed disparity of pneumonia severity by sex in the hospitals. There are other social factors that may contribute to the observed sex-based disparity in the severity of pneumonia in the hospitals, including household living conditions, pre-existing medical conditions, pattern of care seeking in the community, and cost of treatment borne by the parents. Further, if female children with similar severity of pneumonia were brought to lower-level facilities instead of the tertiary level facilities, the disease severity and outcomes observed in the study hospitals may not be a true reflection of the severity of illness in the majority of the hospitals in Bangladesh [60]. Hence, we cannot establish a sex-based disparity in children, but instead, perhaps have generated indirect evidence of sex-based disparity in care seeking for pneumonia at the hospitals.

Despite these limitations, we observe that females are hospitalized less in this study setting, which is consistent with findings from rural Bangladesh [20]. Moreover, the national data documents that death is more common in female children in the community in Bangladesh [61], and our study suggests that deaths related to pneumonia may be a contributor. Further characterization of the sex-based differences in the incidence of pneumonia in the community and the barriers to utilization of the available community health services, particularly at the earlier stages of pneumonia, would be important to assess the true impact of sex as related to the observed outcomes. Estimation of sex disparity in pneumonia incidence, severity of illness, and utilization pattern of health facility by parents for pneumonia should be investigated together in order to exclude parental preference for hospitalization of males over females for treating pneumonia in hospital.

This study has generated evidence about severity and outcomes of pneumonia in children within hospitals in Bangladesh prior to respiratory vaccines being introduced into the country’s childhood immunization program. Thus, it creates an essential baseline measure for assessing the impact of the vaccines on the burden and severity of pneumonia in hospitals in Bangladesh in the future. Bangladesh has introduced Hib vaccines in the routine child immunization program in 2009 and completed full-scale implementation countrywide by 2011 [62]. The pneumococcal vaccine has been introduced in 2015, with full-scale implementation countrywide projected to be completed in a few years [63], and this implementation may help to reduce the risk of severity of illness in children under five years in Bangladesh. A periodic observation of hospital data generated through a systematic surveillance would support monitoring the success of the ongoing interventions and contribute to recommending policies for improved child survival in Bangladesh. As such, this report creates compelling evidence for further investigating disparities by sex in hospital outcomes for pneumonia in other developing countries in South Asia.

## Conclusion

This study demonstrates that female children have more severe pneumonia at the time of admission to tertiary care hospitals in Bangladesh and the risk of a fatal outcome from pneumonia is higher in female children than in males. It is essential to conduct community-based studies to better understanding disparities of pneumonia incidence and severity by sex. Investigations into household factors that may lead to parental preferences for seeking care for their children with pneumonia may provide a deeper understanding of the role of sex in pneumonia outcomes in hospitals in Bangladesh.

## Supporting information

S1 Table Pattern of management of pneumonia and outcome in hospital by sex. (DOCX)

S2 Table Factors contributing to fatal outcome of pneumonia in hospital (Bivariate analysis-odds ratio). (DOCX)
