# Supplementary material for: Disparities by sex in care-seeking behaviors and treatment outcomes for pneumonia among children admitted to hospitals in Bangladesh
Source: PLoS One. 2019 Mar 7;14(3):e0213238. doi: 10.1371/journal.pone.0213238 (PMC6405050; doi:10.1371/journal.pone.0213238)
Supplement: S1 Table — (DOCX) [file pone.0213238.s001.docx]

**S1 Table . Pattern of management of pneumonia and outcome in hospital by sex**

| **Variable** | **Total**  **(N=6856)** | **Female (N=2,401)** | **Male**  **(N=4,455)** | **P value^*#^** |
| --- | --- | --- | --- | --- |
| Antibiotic taken, n (%) | 6711(97.9) | 2363(98.4) | 4348(97.6) | 0.02^*^ |
| Overall oral drug taken (syp. ,suspension ) | 505(7.4) | 183(7.6) | 322(7.2) | 0.54 |
| Overall Injectable drug taken | 6261(91.3) | 2216(92.3) | 4045(90.8) | 0.03^*^ |
| Non severe pneumonia | 1016(16.2) | 353(15.9) | 663(16.4) | Ref. |
| Severe pneumonia | 3939(62.9) | 1364(61.6) | 2575(63.7) | 0.94 |
| Very severe pneumonia | 1306(20.9) | 499(22.5) | 807(20) | 0.08 |
| Supportive Measure | 6716(98%) | 4357(63.5) | 2359(34.4) | 0.46 |
| Nebulization, n (%) | 5118(76.2) | 1779(75.4) | 3339(76.6) | 0.26 |
| Bronchodilator, n (%) | 3298(49.1) | 1131(47.9) | 2167(49.7) | 0.16 |
| O2 inhalation | 4405(65.6) | 1561(66.2) | 2844(65.3) | 0.46 |
| Antipyretic ^a^ | 4480(66.7) | 1596(67.7) | 2884(66.2) | 0.22 |
| Anti-convulsent ^b^ | 216(3.2%) | 72(3.1) | 144(3.3) | 0.57 |
| Special Care required | 305(4.5) | 119(5.0) | 186(4.3) | 0.14 |
| Doctors treated children as pneumonia | 3890(56.8) | 1400(58.3) | 2490(56) | 0.05 |
| Positive culture report | 314(4.6) | 117(4.9) | 197(4.4) | 0.39 |
| Hib ^c^ | 5(1.6) | 1(0.9) | 4(2.0) | 0.43 |
| *S.pneumoniae* ^d^ | 11(3.5) | 5(4.3) | 6(3.0) | 0.56 |
| Length of Stay in the hospital, Mean(SD) | 5.1(3.5) | 5.21(3.89) | 5.06(3.23) | 0.09 |
| Children discharged by doctors after improvement, *n (%)* | 5560(81.1) | 1926 (80.0) | 3634(82.0) | 0.18 |
| Children referred to other facilities for better management, *n (%)* | 40 (0.6) | 25(0.6) | 15(0.6) | 0.74 |
| Children died in hospital after admission, *n (%)* | 276(3.6) | 113(4.8) | 163(3.6) | 0.04^*^ |
| Children left hospital against medical advise | 986 (14.1) | 343(14.3) | 625(14.1) | 0.12 |

*: Z-test to compare difference of two proportions; Statistical significance at p<0.05

97.9% of the children taken antibiotic; proportions of the antibiotic taken was higher among female than male

^a,b^ 140 were missing sample and the denominator was 6716.

^c,d^ The denominator was 314(Male :197 and Female: 117)

#: Independent sample t-test for mean difference
