# Supplementary material for: Disparities by sex in care-seeking behaviors and treatment outcomes for pneumonia among children admitted to hospitals in Bangladesh
Source: PLoS One. 2019 Mar 7;14(3):e0213238. doi: 10.1371/journal.pone.0213238 (PMC6405050; doi:10.1371/journal.pone.0213238)
Supplement: S2 Table — (DOCX) [file pone.0213238.s002.docx]

**S2 Table. Factors contributing to fatal outcome of pneumonia in hospital (bivariate analysis-odds ratio)**

| **Variables** | **Total**  **(N=6856)** | **Outcome** | | **Unadjusted OR**  **(95% CI)** |
| --- | --- | --- | --- | --- |
|  |  | **Fatal**  **(276; 4.0%)** | **Non-fatal**  **(6580; 96.0%)** |  |
| Sex |  |  |  |  |
| Female | 4455(64.1) | 113(40.9) | 2288(34.8) | 1.30(1.02-1.66)^*^ |
| Male | 2401(35.0) | 163(59.1) | 4292(65.2) | Ref. |
| Child age category |  |  |  |  |
| Infant (2-11 month) | 4871(71.0) | 226(81.9) | 4645(70.6) | 1.88(1.38-2.57)^#^ |
| Older (12 +month) | 1985(28.1) | 50(18.1) | 1935(29.4) | Ref. |
| Father education, n(%)^c^ | | | | |
| Complete >10 yrs of schooling | 712(10.4) | 16(5.8) | 696(10.6) | Ref. |
| Did not complete >10 yrs of schooling | 6136(89.6) | 260(94.2) | 5876(89.4) | 1.93(1.16-3.21)^*^ |
| History of antibiotic prior hospitalization | | | | |
| Yes | 3077(56.4) | 108(54.3) | 2969(56.5) | Ref. |
| No | 2381(43.6) | 91(45.7) | 2290(43.5) | 1.09(0.82-1.45) |
| Parents brought children to hospital | | | | |
| Without consulting with a doctor | 3217(46.9) | 164(59.4) | 3475(52.8) | Ref. |
| After consulting with a doctor | 3639(53.1) | 112(40.6) | 3105(47.2) | 1.31(1.02-1.67)^*^ |
| Severe Malnutrition’s (waz <-3SD) | |  |  |  |
| Yes | 1810(26.7) | 142(54.8) | 1683(25.8) | 3.25(2.74-4.53)^#^ |
| No | 4966(73.3) | 117(45.2) | 4834(74.2) | Ref. |
| Hospital admission | | | | |
| Government | 4031(58.8) | 165(59.8) | 3866(58.8) | 1.04(0.82-1.33) |
| Private | 2825(41.2) | 111(40.2) | 2714(41.2) | Ref. |
| Blood culture | | | | |
| Positive | 314(4.6) | 30(10.9) | 284(4.3) | 2.70(1.82-4.02)^#^ |
| Negative | 6542(95.4) | 246(89.1) | 6296(95.7) | Ref. |
| Injectable drug taken | | | | |
| Yes | 595(8.7) | 271(98.2) | 5990(91) | 5.34(2.20-12.98)^*^ |
| No | 6261(91.3) | 5(1.8) | 590(9) | Ref. |
| Severity of illness | | | | |
| Very severe pneumonia | 1367(19.1) | 182(65.9) | 1185(18) | 8.82(6.82-11.4)^*^ |
| Others | 5489(80.1) | 94(34.1) | 5395(82) | Ref. |
| Doctors’ diagnosis at discharge from hospital | | | | |
| Pneumonia | 3890(56.8) | 142(51.4) | 3748(57.1) | 0.79(0.62-1.01) |
| Other than pneumonia | 2955(43.2) | 134(48.6) | 2821(42.9) | Ref. |
| Length of hospitalization (LOS) | | | | |
| Median (IQR) | 4(3,7) | 5(3,10) | ---- | 1.14 (1.04-1.12)^*^ |

^a^ child age 2-11 month

^c^ Data missing for 9 children

Statistical significance at p<0.05^*^, p<0.001^**^
